# Supplementary material for: A Bayesian framework for the detection of diffusive heterogeneity
Source: PLoS One. 2020 May 7;15(5):e0221841. doi: 10.1371/journal.pone.0221841 (PMC7205219; doi:10.1371/journal.pone.0221841)
Supplement: Attachment — Submitted filename: Response to Reviewers.pdf [file pone.0221841.s001.pdf]

This study describes a Bayesian inference algorithm to estimate local values of the diffusion coefficient inside live cells from single trajectories of intracellular particles. This type of algorithm can be useful to researchers interested in quantifying diffusivity of heterogeneous or time-varying environments, including but limited to the cytoplasm of live cells. The manuscript describes related existing efforts in the literature, and makes a convincing point that the present algorithm, and in particular its associated freely accessible implementation, is sufficiently different from those existing efforts. In particular, while the parametric nature of the present study is a limitation with respect to existing, non-parametric, efforts, its simplicity may be advantageous to those without significant expertise in statistical mechanics.

> We'd like to thank the reviewer for their thoughtful consideration of our manuscript and helpful comments. Indeed, we hope that this tool may be of particular use to researchers without deeply computational or statistical backgrounds, offering a modifiable tool with more flexibility than the MSD, but less complexity than existing software that can have a higher barrier to entry.

The manuscript analyzes the error in the estimated D based on the localization error of the particle. As expected, the error decreases with the length of the recorded trajectory. However, Figure 5 shows this error seems to be unacceptably large for some combinations of values, and it is unclear whether a "typical experiment" (this is a loose term whose meaning is expanded below) would yield acceptable results. The authors argue that the purpose of Figure 5 is for each researcher to assess the error for their own experiments. This is valuable but Figure 5 is plotted in a way that makes this assessment difficult:
1) Only two values of D are covered. It would seem to make more sense to plot Figure 5 normalizing the localization error with (D*tau)^(1/2). This could capture the D-dependence of the estimation error, and only one panel might be necessary to cover all D values.
2) Second, a line plot or contour plot format would be preferable to read errors in the plot.

> I agree with the reviewer that this kind of non-dimensionalization would offer a more robust representation of the model results. There are many relevant parameters whose relative values impact the estimation error, and careful choice of how to represent this data is certainly important. However, since we are aiming for this tool to be engaging for a more experimental audience, we wanted to maintain the use of more tangible parameters, which maintain their straightforward physical interpretability, and chose this format to prioritize familiarity and approachability over density of information reporting.
>
> For any given experiment you may have a single localization error and limited range of trajectory lengths. So understand why the parameterization of this figure may not be the most versatile in its use for any one experiment. However, we hope that this parameterization of the lookup table might instead be applicable to a wider audience. As this figure is viewed by a wide audience of researchers with trajectories of varying lengths and localization errors constraining their experiments, we hope they can get a ballpark answer for the question of whether this tool will be useful for them. While the diffusion coefficient value is of course relevant as well, we hope that representing the effects on three different orders of magnitude of diffusivities can give the reader a taste of what might be possible (a third order of magnitude was added in our revised manuscript).

That said, the tool is designed to have an accompanying tutorial Jupyter notebook with examples of how each figure is generated. This was a conscious choice to ensure that those without extensive computational backgrounds have a more easily approached interface with the code, in order to tweak analysis parameters themselves, and see how the results change if they switch out parameter values to generate adaptation of our provided figures which are tailored to their own experimental parameters and constraints. We hope that this design can help make up for the limitations in what can be presented in the manuscript figures.

3) It would be informative to represent a “typical experiment” or experiments in the localization error and trajectory length coordinates of Figure 5. The authors can use experiments from the literature and / or their own data from previous studies.

Thank you for this feedback - we have incorporated this suggestion.

As the authors point out, a limitation of the study is that it focuses on an idealized model of intracellular diffusion. The authors argue that the method could be adjusted to account for complicated phenomena, such as subdiffusion, but the intended audience of this algorithm may not find this straightforward. This issue is compounded with the fact that this reviewer finds the purely diffusive case to be particularly amenable to the analytical calculation of the posterior distribution. Other cases may be harder… It would be informative to illustrate how the algorithm would be modified in the subdiffusive or e.g., persistent-random walk case by presenting the posterior distribution for those processes.

Finally, there may be cases in which modifying the algorithm to account for non-purely diffusive, isotropic behavior is not feasible or where the actual behavior that needs to be accounted for is unknown a priori. It would be informative to know the error in estimated D in those cases. Again, subdiffusive, persistent-random or anisotropic random walk cases come to mind.

We agree that demonstrating example adaptations to the prior and posterior for more complex intracellular would be an exciting enhancement of what this manuscript could offer, however these advancements are beyond the scope of what we are hoping to explicitly provide within this manuscript. We hope that the detailed demonstration of how this tool is built and acknowledgement that prior and posterior distributions may be adapted for more complex needs is sufficient for demonstrating the value of this tool.

However, we agree with the reviewer’s important note that sub-diffusive motion is of particularly great importance to address in greater detail. To address this important concern, we have taken the reviewers suggestion of applying our analysis tool (with the existing prior and posterior designed for pure diffusion) to biologically- relevant sub-diffusive trajectories and reported the resulting estimation error. For this task we have used trajectories simulated using fractional Brownian motion, as previous work has shown this to be a prevalent mode of intracellular transport. The Hurst coefficient (H) used to define this process is H = alpha/2, where alpha is the parameter giving the MSD’s scaling with time lag (tau) as in Eq 1 of our manuscript. Thus, we have set the Hurst coefficient in the simulated trajectories to best represent reported results for the sub-diffusive time scaling alpha = 0.75 (or H = 0.375).

We feel this was an important addition to the manuscript in demonstrating the applicability of the tool and are grateful for the reviewer's suggestion to include this.

Minor comments:

1) Is the alpha in equation 2 related to the alpha in equation 1?

Thanks for pointing this out! No, the two are not related. We've changed the inverse gamma parameters to (a, b) rather than (alpha, beta) to disambiguate.
