# Supplementary material for: A Bayesian framework for the detection of diffusive heterogeneity
Source: PLoS One. 2020 May 7;15(5):e0221841. doi: 10.1371/journal.pone.0221841 (PMC7205219; doi:10.1371/journal.pone.0221841)
Supplement: Attachment — Submitted filename: response_to_reviewers.pdf [file pone.0221841.s002.pdf]

Reviewer #1: I appreciate the authors' efforts to address my concerns and am for the most part satisfied with their revisions. I have a couple of remaining comments.

Thanks for taking the time to consider our manuscript again and for your helpful feedback.

First, the list of references is rather short and there are places at which the authors discuss standard statistical inference theory without providing appropriate references to the literature. Perhaps a graduate level textbook would be enough. I believe this would be important considering the targeted audience.

We appreciate the suggestion and have added references to a graduate Bayesian statistics text (Gelman et al's "Bayesian Data Analysis") where appropriate.

Second, I still believe the authors overestimate the generality / flexibility of their approach. I understand the computational framework they present could be extended to other scenarios more representative of intracellular fluctuations than a Gaussian process. However, it is not clear that these extensions would be trivial. In fact, they even recognize this point themselves (circa line 370). I appreciate the authors including a section where they benchmark their tool for fractional Brownian motion. I would suggest to temper the statements about generality of the framework.

We have removed the "flexible" descriptor in line 354 and added another statement acknowledging the challenge of framing more complex priors/ posteriors in line 372-374 in the "Framework limitations" sections.

Also, please use the same color axis and color bars in figures 5 and 7 to facilitate direct comparison (as in by caxis of Matlab or clim of python).

Thanks for catching this; we've updated the rendering of these figures so that each comparable plot (ie 5A/7A, 5B/7B etc have identical color axes / color bars.

Reviewer #2: The authors manuscript with the accompanying, well-

documented python repository is a valuable tool for researchers without significant expertise in Bayesian statistics.

We appreciate you taking the time to review our manuscript and accompanying repository.

It would be more helpful to gather better intuition for KL divergence criterion with more details on how to interpret Fig 6. For e.g, an approximate threshold value of threshold KL below which the posteriors have a given probability to represent the same true diffusion constant (and therefore, not representative of the heterogeneous environment). Authors explain the intuition of KL values, but a rule of thumb would be more beneficial to design experiments.

Thanks for this suggestion; we agree that including a benchmark value would increase the usability of this reference table and have included a value and associated brief discussion in lines 332-342.

The authors also provide an accessible way of estimating baseline errors in inference using heatmaps in Fig 5. A very important source of sensitivity to parameter inference lies in prior distribution parameters and a brief guide of choosing parameters (a,b) to not introduce bias in analysis (uninformative prior) would be recommended.

We agree that a discussion of this prior bias and parameter choice strengthens the manuscript and the tool's usability; we've included a brief discussion of this in lines 191-208.

Minor typo: In pg 7/18, the likelihood function is written as p(theta I x) instead of p(x I theta).

Thanks for catching this typo; we've fixed it in the updated manuscript.
